# Where is the pain? A qualitative analysis of Ghana’s opioid (tramadol) ‘crisis’ and youth perspectives

**DOI:** 10.1371/journal.pgph.0001045

**Published:** 2022-12-21

**Authors:** Jacob Albin Korem Alhassan

**Affiliations:** 1 Ad Astra Foundation, Tamale, Ghana; 2 Oxford School of Global and Area Studies, University of Oxford, Oxford, United Kingdom; 3 Department of Community Health & Epidemiology, University of Saskatchewan, Saskatoon, Canada; Brigham and Women’s Hospital, UNITED STATES

## Abstract

Over the last five years, media reports in West African countries have suggested a tramadol abuse ‘crisis’ characterised by a precipitous rise in use by youth in the region. This discourse is connected to evidence of an emerging global opioid crisis. While the reported increase in tramadol abuse in West Africa is likely true, few studies have critically interrogated structural explanations for tramadol use by youth. Nascent academic literature has sought to explain the rise in drug use as a function of moral weakness among youth. This Ghanaian case study draws on primary and secondary data sources to explore the pain that precedes tramadol abuse. Through a discourse analysis of 295 media articles and 15 interviews (11 with youth who currently use tramadol and 4 with health system stakeholders), this study draws on structural violence and moral panic theories to contribute to the emerging literature on tramadol (ab)use in West Africa. The evidence parsed from multiple sources reveals that government responses to tramadol abuse among Ghanaian youth have focused on arrests and victim blaming often informed by a moralising discourse. Interviews with those who use tramadol on their lived experiences reveal however that although some youth use the opioid for pleasure, many use tramadol for reasons related to work and feelings of dislocation. A more complex way to understand tramadol use among young people in Ghana is to explore the pain that leads to consumption. Two kinds of pain; physical (related to strenuous work) and non-physical (related to anxiety and the condition of youth itself) explain tramadol use requiring a harm reduction and social determinants of health approach rather than the moralising ‘war on drugs’ approach that has been favoured by policy makers.

## 1. Introduction

**Fig 1:**
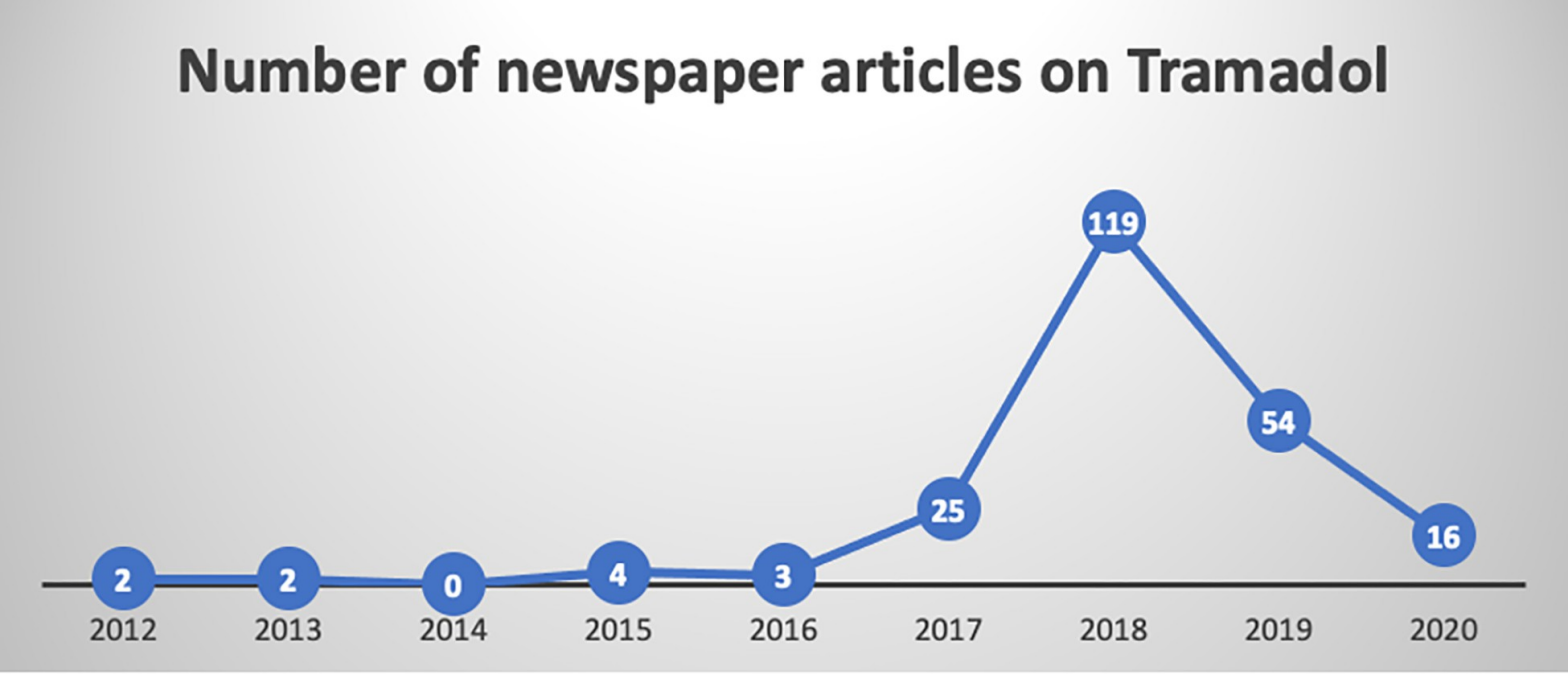
Evolving trends in newspaper reports on tramadol in Ghana. Source: Author.

“It is important we let people know that abusing medicines like tramadol can have bad consequences including death” [1]. These words by the Chief Executive Officer (CEO) of Ghana’s Food & Drugs Authority (FDA) were published in an article on 5 May 2018 on *GhanaWeb*, one of Ghana’s main online news outlets. Although a couple of years prior most newspaper articles did not routinely mention tramadol or its popularly contracted form “tramol” in their reportage (see Fig 1), by 2018 it had become common to see newspaper publications and radio and television programs dedicated to helping solve the tramadol ‘crisis’. In the ensuing months international news outlets such as *Deutsche Welle* all pointed to a rising trend in the consumption of “cheap and accessible” opioids by Ghanaian and West African youth [2]. While the current West African situation was linked to tramadol, it must be understood in the context of more complex histories of drug use in Africa [3] and a rise in opioid use globally driven by multiple factors ranging from “aggressive marketing” [4] by pharmaceutical industries to changing criminal networks connecting and distributing drugs across continents [5]. The opioid crisis is increasingly recognised as a global health issue particularly in North American and European contexts where pharmaceutical companies are beginning to be held accountable for decades of over-prescribing opioids while understating the dangers of opioid use [6].

Reports of precipitous increases in drug use have become common across several African countries for different drugs. In the context of the COVID-19 pandemic for example, associated joblessness has led to a rise in drug use in countries such as Zimbabwe where more youth use crystal meth [7], while recent research evidence from South Africa illustrates increasing heroin use among the young male labouring poor, many of whom have precarious employment and use heroin as a coping mechanism [8].

This article is situated in the broader context of drug use in Africa, discourses of a dangerous and precipitous rise in drug use, especially among youth, and questions on how to *explain* this issue. In this article, I explore the reported rise in tramadol consumption and use in Ghana and media portrayal of this phenomenon over the last five years. I argue that the framing of the tramadol ‘crisis’ was a moral panic and that such framings helped justify the solutions the government and key stakeholders have adopted to respond to its use—namely, public education and arrests while ignoring root causes of drug use such as poor economic prospects. Relying on the lived experiences of Ghanaian youth who use tramadol, I draw on rich local descriptions to offer a different way of thinking about these youth, as many are simply young people striving to survive in an economy where their situations are highly precarious.

Drawing on key themes in Africanist scholarship, I contribute to this nascent literature by critically (re)interpreting the rise in tramadol use in Ghana in the context of youth and waithood. I argue that its use needs to be understood in the context of the physically demanding and precarious employment conditions many young Ghanaians face, their anxiety and uncertainty regarding their prospects and the demanding conditions of work with which many of them must contend.

Finally, I argue that a better way to understand tramadol use among young people in Ghana is to explore the *pain* that leads to consumption while moving away from the moralising discourses that have been the core approach of the government and other stakeholders. By paying particular attention to pain and themes of youth—and through a proposed framework—I show that tramadol is used as a coping mechanism for many young people who are struggling to find their place in the context of perpetual waithood and global neoliberal processes that shape young people’s lives [9]. The reality of tramadol use requires improvement of young people’s economic prospects and strengthening of mental health systems rather than victim blaming.

### 1.1 Tramadol in Ghana

Tramadol is a pharmaceutical drug with weak μ-opioid agonist properties, whose M1 metabolite is the primary mechanism for its analgesic (pain killer) effect [10]. The drug was originally distributed by the German pharmaceutical company Grünenthal in 1977. It was historically used to treat pain in situations where paracetamol, non-steroidal anti-inflammatory drugs (NSAIDs), and cyclooxygenase (Cox)-II inhibitors were insufficient or in patients without tolerance to these alternatives [11]. It is the only opioid not under international drug control. Multiple meetings (four between 1992 and 2006) by the World Health Organisation Expert Committee on Drug Dependence (ECDD) concluded that tramadol has low abuse potential especially among those with no history of substance use although research from China showed some evidence of abuse potential [12].

The emerging academic literature on tramadol in Ghana falls into two main categories. (There is a third emerging literature on the role of the media in the tramadol ‘crisis’; see Thompson and Ofori-Parku) [13].

First, there is a developing body of epidemiological literature describing patterns of tramadol use. In a study by Elliason and colleagues [14] in the Wassa Amenfi West Municipality in the Western Region, consumption patterns were compared by socio-demographic characteristics. Among those sampled the study found that 45% of those who consume tramadol were male and 49% were aged 16–30. One of the most interesting findings from the study which did not receive much analytical attention was the fact that only two per cent (2%) of those who consume tramadol worked in the ‘formal sector.’ Saapiire et al. [15] based on research in Jirapa in northern Ghana also recently reported a high prevalence of tramadol abuse especially among males (90.7%) compared to females (9.3%) and concluded that formal sector employment is “protective against tramadol abuse.” Given the high percentage of usage among young men working in the informal sector, it would be useful to explore why this demographic uses tramadol more and the ways the context of work may be implicated.

The second body of published academic literature on tramadol has been qualitative studies exploring facilitators and motivations for use. The qualitative literature describes some of the context of this use and primarily focuses on motivations for use and effects of tramadol on users. Fuseini et al. [16] concluded for example that peer pressure, curiosity and post-traumatic addiction are the main initiating factors for tramadol use. This study also argued that effects of consumption can be desirable (analgesic psychological effects and even aphrodisiac effects) or undesirable (vomiting and seizures or irritability and aloofness) [16]. Finally, in a qualitative study in Kumasi, Peprah and colleagues [17] also drew similar conclusions regarding the drivers of tramadol consumption, arguing that people use it because of sexual, psychological and physical motivations.

There are some gaps in the emerging tramadol literature and opportunities for further research. First, most of the literature focuses on the *effects* of consumption rather than structural causes of tramadol use. Additionally, academic publications on effects of tramadol use have highlighted key challenges including suicide ideation [18] although almost no overdoses have been reported from Ghana unlike has been the case with opioid crises elsewhere [4]. The absence of academic reports on overdoses could be because of the lower potency of tramadol although it could also be a function of data unavailability in the African context [19]. In countries such as the Islamic Republic of Iran for example, overdose rates from tramadol are high and a common cause of poisoning admissions to emergency departments [19]. Given these dynamics, as the tramadol situation continues to evolve in Ghana, there might be potential lessons for other contexts where stigma related to opioid use has been connected to high overdose rates. Moreover, although extant studies have described the demographic characteristics of tramadol users and highlighted high usage among men, informal sector workers and youth, they have done so mainly through descriptive statistics. This necessitates more research on the lived experience of youth who use tramadol. Finally, there is an emerging interest in harm reduction in Ghana -in the form of needle exchange programs, HIV and hepatitis B & C prevention strategies [20]—as has been advocated for in Europe and North America. The fact that tramadol is predominantly orally ingested means that new approaches to harm reduction will need to be envisioned drawing on the lived experiences of those who use tramadol.

Drug use is always influenced by a complex set of factors including “productivity, pleasure and intimacy” [21] thus a biopsychosocial understanding demands a delicate balancing of the deeper structural factors that construct the world inhabited by those who use drugs with their complex lived experiences [22]. This study turns the analytical gaze toward *pain*. Such attention reveals the broader causes of tramadol use because attention to and analysis of pain reveals “how the bodily experience itself is influenced by meanings, relationships, and institutions” [23].

## 2. Theoretical orientations

This article draws on multiple theories to problematise ongoing framings of tramadol use. First, it draws on the notion of a ‘moral panic’, a state that occurs when “a condition, episode, person or group of persons emerges to become defined as a threat to societal values and interests” [24]. As applied to youth in Africa, the notion of moral panic is of particular importance given the historical and ongoing representation of African youth as “unruly, destructive and dangerous forces needing containment” [25]. In practical terms, throughout the article and in analyses, references to tramadol use by youth as “immoral” or a “crisis” are treated with some scepticism while assuming that particular framings of young people as irresponsible drug users can be used by those in power to deflect attention from root causes of drug use [26].

Secondly, the article draws on anthropological theories of structural violence and the idea of the social determinants of health. The concept of ’structural violence’ was first used by Galtung [27] to differentiate violence that is direct or interpersonal from violence that is built into social structures. Galtung’s original article was in the field of Peace Studies, however this idea has subsequently been used by authors across disciplines to explore how social structures reduce people’s life chances. For medical anthropologist Paul Farmer [28], structural violence is “violence exerted systematically” and to study this is to explore the “social machinery of oppression.” Understanding health issues such as addiction therefore requires attention to the ‘pathologies of power,’ the structures that force people to engage in health-destructive behaviours [29]. Many researchers have used this approach to understand the health of marginalised groups such as Latino migrant workers in the USA [30] or the drug economy in deindustrialised urban contexts [31]. I reinterpret tramadol use by Ghanaian youth in the context of the broader social structures of oppression in which they live and work. I draw as well on the idea of the social determinants of health [32] which posits that health behaviours are influenced by social structures. Thus, where problematic drug use emerges in the analysis, I emphasise the importance of harm reduction principles and addressing the living conditions of youth as an essential step in improving the lives of those who use tramadol.

## 3. Methods of the study

### Ethics statement

This study received ethics approval from the University of Oxford Social Sciences and Humanities Interdivisional Ethics Committee (SSH_OSGA_ASC_C1_21_030). The study also received an ethics exemption under the Ghana Health Services Ethics Review Committee Standard Operating Procedures 2015 given that it involved interviews with voluntary participants who gave informed consent. Informed consent was obtained from participants verbally and recorded before each interview. Three affirmations were used to secure informed consent. Each participant confirmed (1) that their participation in the research was voluntary, (2) that they give their verbal consent to participate, and (3) that a voice recorder could be used to record responses. The 11 participants received a $10 honorarium each.

### Study design

This study is part of a broader research project to understand the drivers of tramadol use among West African youth. I report here mainly on the newspaper sources with some references to interview data although a longer discussion of lived experiences of youth who use tramadol is reported elsewhere [33]. The research employed a qualitative case study methodology [34] drawing on multiple methods and relying on primary and secondary data sources. The secondary data analysis involved a discourse analysis [35] of media reportage on tramadol use in Ghana between 2012 and 2020. In total, 295 newspaper articles were systematically extracted from newspaper search site *Factiva*, imported into NVivo 12 software, and inductively coded to describe discourses on tramadol consumption (see Fig 1. for trends in reportage and S1 Table for codes). To conduct the discourse analysis, each article was read in its entirety and coded for: 1) how it described those who use tramadol; 2) explanations offered for tramadol use; 3) descriptions of the effects of tramadol use; and 4) how each article framed the solution to the problem of tramadol use. Once this process was completed codes were combined to explore patterns and to describe the dominant frames used in writing about the tramadol crisis [35].

Additionally, primary qualitative interviews (lasting between 30 min. and 1 h 15 min.) were conducted with fifteen (15) individuals between March and May 2021 via telephone due to the COVID-19 pandemic. Interviews were transcribed verbatim and imported into NVivo 12 software. The final sample of 15 participants was considered appropriate based on emerging analysis that revealed similar responses across interviews (approaching saturation) and the unpredictable pandemic context where interview data collection could only be performed at a distance. Recruitment was done through purposive sampling after a research poster was circulated on social media. Other participants were recruited through personal contacts given the sensitive nature of the research topic. Interviewees consisted of eleven (11) people (ten males and one female) who were currently using tramadol and worked in diverse professions (tricycle riders, self-identified ‘hustlers’, students, teachers, masons etc.). For these 11 participants the criteria for inclusion was that they be youth (age 15–35) who used tramadol [36]. Interviewees included in this analysis were aged 19–35 and had used tramadol over the last year. Interviews were conducted in English, Dagbani and pidgin English using a semi-structured interview guide. Interviewees were asked to provide a short life history, describe what led them to tramadol use, highlight socioeconomic factors that explain why they use tramadol and describe societal views of those who use tramadol. They were also asked to comment on any available supports if they wanted to stop using tramadol and finally, they were asked to share their hopes for the future (S1 Text).

The four other interviews were conducted with stakeholders (a journalist, a mental health nurse, a program manager and a divisional head of one of Ghana’s drug regulatory bodies) who had worked on tackling the problem of tramadol use. These stakeholders were also purposively recruited through a research poster on social media and via personal contacts to better understand Ghana’s policy response to tramadol use. The stakeholders had played keys role in the response to tramadol use in Ghana and provided high-level explanations of how they as individuals and members of institutions understood the problem and the solutions they were advocating to respond to the ‘crisis’.

Participants were recruited across cities (Accra, Tamale, Kumasi and Winneba). Illustrative quotes are labelled according to the location and sequence of interviews: T1-T4 designate Tamale-based interviews; K1-K4 (Kumasi); A1 (Accra), W1-W2 (Winneba), HCP1-3 (Healthcare Professionals), BJ (Broadcast Journalist).

To complete interview data analysis all transcripts were imported into NVivo 12 software and analysed using the six-step process described by Braun and Clark [37]. This involved: 1) familiarisation—transcripts were read and re-read; 2) generating initial codes—interesting features of each transcript were coded systematically in NVivo; 3) searching for themes—codes were combined into categories and collated into potential themes; 4) reviewing themes—emerging themes were compared to coded extracts; 5) defining and naming themes—themes that told the overall story of the analysis were refined; and 6) producing the report—themes were written up. Emerging findings were discussed with four participants who provided feedback. Research findings are reported drawing on COREQ guidelines (S1 Checklist) [38].

### Reflexivity

I approached this study from a critical ontological perspective as a PhD trained health researcher with about 5 years of experience with qualitative methodology. My methodological choices were guided by a commitment to reveal the role of power in shaping vulnerability. I drew on my Ghanaian identity to build ethical and respectful relationships with participants. I recognise that my positionality shapes my framing of the research project and endeavoured during interviews to avoid the use of jargon and to meet participants at a level of equality. I spoke in pidgin and my local language (Dagbani) where necessary and adhered to key principles of relational ethics in working with interviewees who used tramadol.

## 4. Findings and discussion

It is not entirely clear what caused a major spike in media reportage on tramadol use in 2018 as shown in Fig 1. The first Ghanaian newspaper reports on tramadol emerged from publications about drug seizures in 2012 in Nigeria. One such article noted that truckloads of drugs intercepted in some ports in West Africa had claimed to be from Ghana as part of the Economic Community of West African States’ (ECOWAS) trade liberalisation scheme but “actually originated from China” [39]. By 2015 several newspapers were regularly publishing articles on tramadol. Some of these articles focused on contracts awarded to companies to provide drugs for the health sector. From this period onwards news reports emerged regularly on tramadol and were often about criminals arrested with tramadol in their possession. The trend continued for a few months and by March 2018, Ghana’s minister of health issued warnings on the dangers of tramadol use. The sections that follow describe how tramadol use was explained and represented by the media vis à vis theoretical literature on youth.

### 4.1 The condition of ‘youth’ and youth futures

In analysing media sources to understand how stories are framed, one of the most salient characteristics of tramadol stories has been their connection to youth. The seventh most common word found in newspaper stories was ‘youth’, used 539 times although never clearly defined. That notwithstanding, youth—however defined—remains an important category for theoretical reasons and requires particular attention since participants themselves often invoked ideas of youth in explaining why they used tramadol.

The concept of youth, although sometimes used as a biological signifier to refer to people within the age bracket of 18–25 [40], is a complex sociological category and subject to manipulation. As Bourdieu [41] argued, across societies definitions of youth are subject to power dynamics and “the frontier between youth and age is something that is fought over in all societies.” This is particularly the case since the concept of youth and its connotations can be manipulated as a means of scapegoating and apportioning blame.

There are several reasons youth is a fertile ground for the study of health in Africa. First, youth are “particularly sensitive to transformations in the economy as their activities, prospects and ambitions are dislocated and redirected” [25]. In this sense, the lived experiences of youth who use tramadol can act as a barometer for understanding structural changes in society. Second, in the African postcolonial context in particular, youth has come to indicate “being disadvantaged, vulnerable and marginal in the political and economic sense” [42]. The fact of marginalisation and disadvantage was described by several interview participants, some of whom described intense despair associated with the condition of youth in Ghana. When I asked one of the interviewees about her hopes for the future as a young person in Ghana she responded:

If I talk about the education side, I really do not have hope because there are a lot of graduates home. And then the maximum of salary some of these graduates get is like 1500 cedis [240 USD] a month. How much is your water bill? Light bill everything? (A1).

Despite such stories of gloom, scholars have sought to explore the condition of youth on the continent not simply as hopeless stories of despair but to recognise within these realities of disadvantage and marginalisation “the diversity of experience as well as the agency and creativity of young people as they try to overcome serious everyday challenges” [43]. In the context of African youth’s continued challenge to governmental and other authorities by seeking to shape their own destinies and create new ‘youthscapes’ [44] and spaces for self-fashioning [45], I start by problematising how the issue of tramadol was framed in media discourses as a problem of ‘youth irresponsibility’. Following this, I explore how such framings fed into the sorts of solutions often proposed by the government and the media. I then contrast this discourse with the lived experience of youth who use tramadol many of whom indicated that palpable pain lay at the heart of their drug use. Interpreted in the context of ethnographies of pain, I argue that tramadol is used to respond to physical and non-physical pain.

### 4.2 Tramadol consumption as individual moral failure among youth

The primary mode of explanation employed by the media to make sense of the rise in tramadol consumption has been that it is abused because of individual moral failings. Several newspaper stories offered a plethora of explanations on why youth may abuse tramadol ranging from senior high school athletes who have “resorted to the abuse of the drug, claiming it enhances their performance” (*All Africa* 4.7.2017) [46] to multiple media stories explaining that “the youth including students also take the drug to exert their strength when having sexual intercourse with their partners while others take it just for pleasure” (*Ghana News Agency* 30.11.2017) [47].

In these morally tinged discourses, the media highlighted that people “knowingly used the drug for recreational and aphrodisiac purposes” (*Ghana News Agency* 8.3.2018) [48]. These discourses were meant to galvanise societal hatred for tramadol users and therefore relied on ideas of promiscuity that would offend the social sense of morality. For example, in an article on drug confiscation in the Ashanti region, an official of the Food and Drugs Authority asserted that “some of the reasons for the abuse of the drug included supposed enhancement of sexual drive and prolonged ejaculation” (*Ghana News Agency* 12.4.2018) [49]. While it might be the case that some people use tramadol for sexual enhancement [17], there was an overemphasis on this dimension. Indeed, most stories were focused either on connecting tramadol to sex or crime. Almost half of the news stories reviewed were about crime and arrests of young people with the drug in their possession. Interestingly, none of the interviewees in this research reported being arrested. Despite the overemphasis on sex, I found through interviews that few people use tramadol *solely* for sexual reasons (only one interviewee mentioned sex as an important reason for his use of tramadol).

Additionally, a neo-traditionalist discourse was invoked to explain tramadol use as a function of moral weakness. There were references by chiefs, queen mothers, religious leaders and opinion leaders who regularly emphasised “the beauty of innocence” and highlighted the necessity for “cultural learning that instils morals, values and norms in the children” as solutions to the tramadol challenge (*Ghana News Agency* 24.11.2018) [50]. According to these authorities, use was on the rise because parents failed to instil morals in their children. The moral discourse often involved the older generation decrying falling moral standards in the country and was so entrenched that even some academic sources relied on this framing as exemplified in Elliason and colleagues [14] who concluded that “the moral upbringing of the youth” is responsible for the tramadol trend.

It is important to state these discourses explicitly because they heavily shaped what were perceived as the solutions to the problem of tramadol use: education or punishment. By framing it as a problem of youth who simply were morally corrupt, authorities often advocated for campaigns to “educate the youth” on the dangers of consumption and the moral imperative to avoid abusing the drug. Regulatory authorities “appealed to parents to advise their children to desist from the use of the drug since it is destroying a greater number of them” (*Daily Guide* 3.8.2017) [51]. When such persuasion failed to yield the desired results a sort of ‘war on drugs’ approach was adopted. The health minister issued an executive instrument on 26 September 2018 (EI 168) banning the sale of tramadol. The instrument also made 250 mg doses illegal although stores could still sell 50 or 100 mg doses. Finally, it was converted to a prescription drug and several newspapers reported on police swoops to arrest youth who continued to use tramadol. Unfortunately, these ‘solutions’ often failed to target some of the underlying root causes of use and most of them also failed to adopt sustainable approaches. For example, during interviews drug regulators indicated that for youth who may have become addicted to tramadol appropriate solutions would have been to offer them access to rehabilitation centres. According to one regulator however these more long-term approaches were not favoured by government. The reason such solutions received less attention is summarised by a mental health nurse who described Ghana’s mental health system inadequacies:

[In Ghana] mental health itself has been relegated to the background to the extent that even the practitioners are stigmatised… How many traditional government facilities do we have in terms of hospitals that respond to the needs of the mentally ill? Only three in the whole country. And two of them are located down south here, Ankaful and Pantang (HCP 3)

Thus, throughout the discussions on tramadol as a problem of youth irresponsibility, authorities failed to understand why the youth use tramadol or to focus on youth-informed structural solutions to tramadol use. In this sense the moralising discourses that have been adopted by the government and other stakeholders have served to obscure the fact that mental health and addiction services are woefully inadequate in the country.

**Fig 2:**
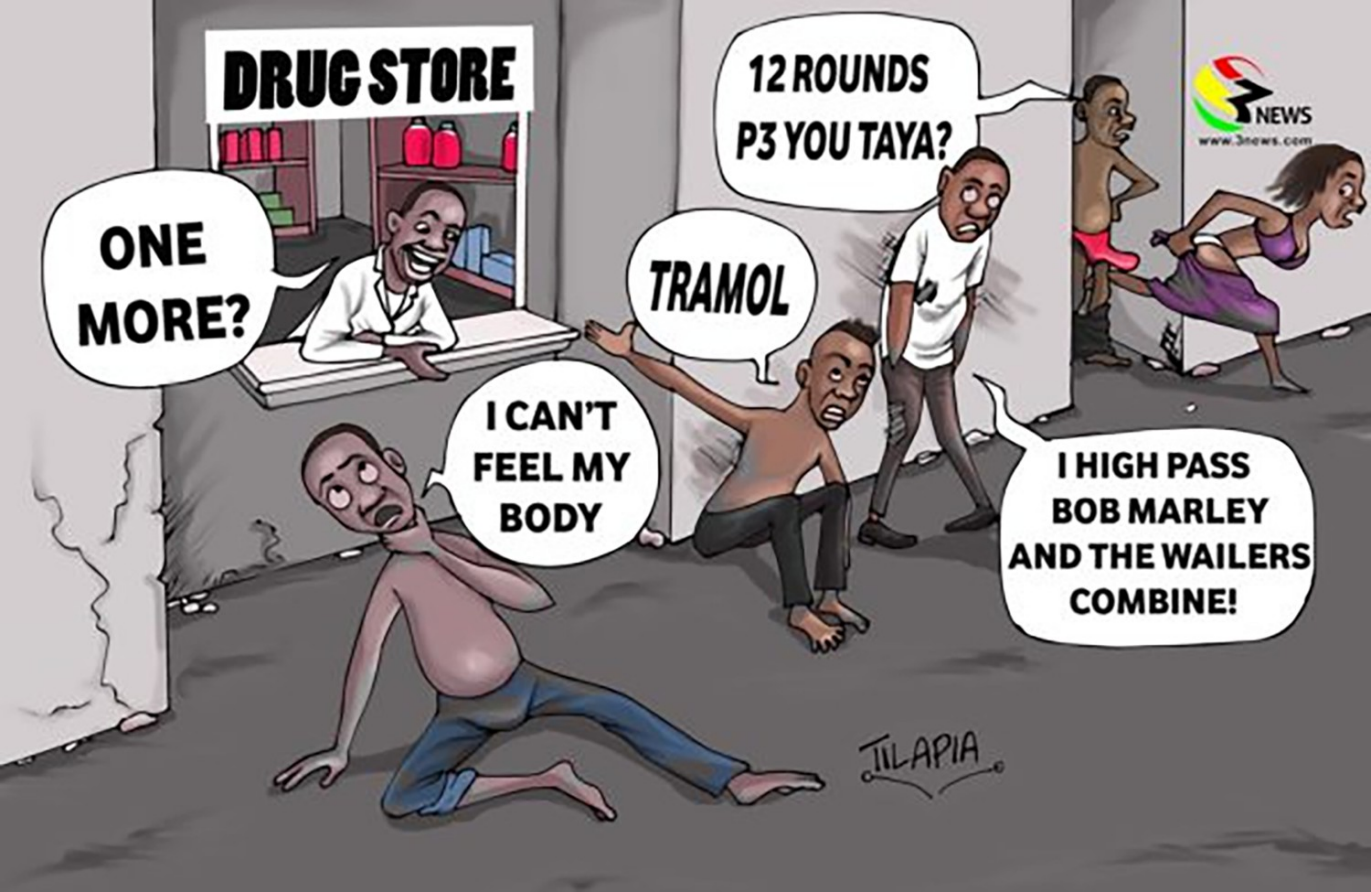
The tramadol sex street. Cartoon by Ghanian artist Tilapia da Cartoonist, April 2018. Reproduced with permission from Tilapia da Cartoonist.

Famous Ghanaian cartoonist, Tilapia, satirically depicted some of the main supposed motivations for tramadol use; namely sex (a woman runs away from a rather virile young man) and feeling ‘high’ (Fig 2). Tilapia also satirically showed how drug store owners laughed through it all as they made money (for example, although the law illegalised 250 mg, users could simply buy multiple 50 mgs to get the same ‘high’). (See: Tilapia, 2018)

### 4.3 Pain as a fundamental reason for tramadol use

The discourses above represent some of the ways the Ghanaian news media have understood and reported on the issue of tramadol use. The reports unfortunately paid less attention to the issue of pain which is problematic since a complex set of factors beyond individual choice often determine drug use and addiction [22]. More importantly discourses that marginalise those who use drugs deserve to be critically assessed because “targeting particular groups of drug users commonly reflect points of social apprehension and serve to enhance the social control and exploitation of subordinated ethnic, class, gender, or other groups characterised as a threat to the status quo” [26]. In the case of tramadol use in Ghana, this insight suggests that one ought to approach some of the media reports with some scepticism. In the remainder of the article, I argue that a fundamental reason young people in Ghana use tramadol is pain, although other important reasons exist. Using this focus, we can humanise those who use it and appreciate the complex contextual drivers of use beyond moral discourses. Attention to pain also serves as a critique of current policy solutions for eradicating tramadol use.

Pain is a fundamental part of the human condition. It has been of interest to scholars in varied disciplines from philosophy to anthropology because it is a “basic existential fact of our distinctly human way of being-in-the-world. To be human is to be vulnerable to both the possibility and inevitability of suffering pain” [52]. Understanding, interpreting and describing someone’s pain thus requires attention to the fact that “a person’s experience of pain is multi-dimensional, relating to culture, emotion, mind and body” [53].

The approach of contextualising pain, while useful, has historically been problematic because anthropological and medical interests in studying it from a cultural perspective sometimes led to the creation of stereotypes. For example, Black slaves and plantation workers were considered impervious to pain because of their supposedly “dulled sensitivities” arising from a less-developed brain [54], while Jews and Italians were described as “tending to exaggerate their pain experience” [55]. Some of these patently racist understandings of pain have unfortunately survived into the present leading to pervasive undertreatment of pain for Black people in settings such as the USA [56].

Despite these unfortunate academic attempts at understanding pain and the negative ongoing effects of such studies, critical accounts exist on the importance of adopting pain as an analytical entry point for exploring individual lived experiences. According to Arthur Kleinman [23] pain “indexes” the “embodiment of cultural categories of distress” and can help us “understand how the bodily experience itself is influenced by meanings, relationships and institutions.” I approached the study of tramadol by trying to ‘elevate’ pain to see what it indexes. During interviews I often asked participants about pain and how it shapes their use. By the end of the research process, it became apparent that many young people in Ghana use tramadol to alleviate two kinds of pain.

**Fig 3:**
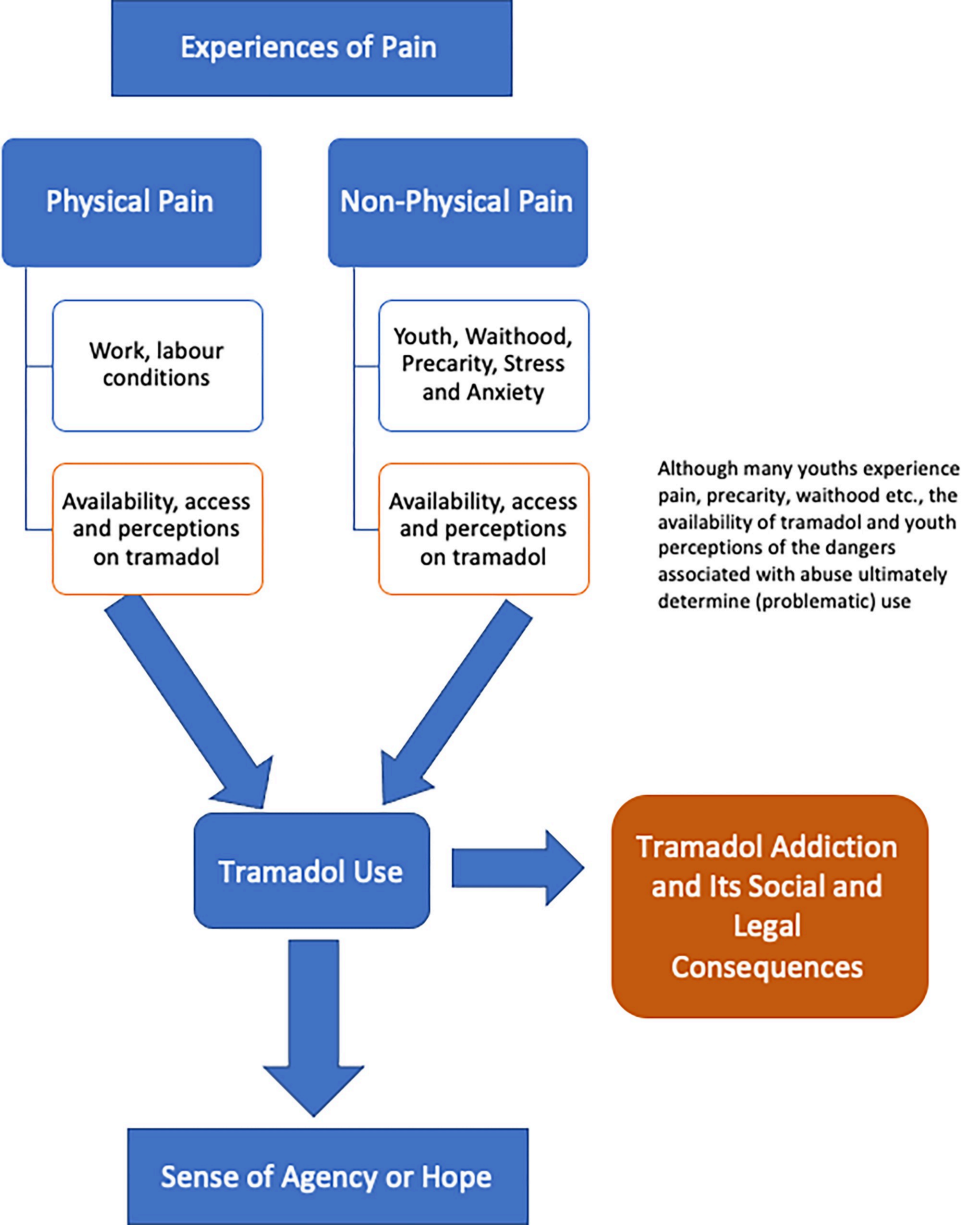
Two kinds of pain. Source: Author.

Unlike the media portrayals of tramadol use as a moral issue, most of the youth I interviewed indicated that they use it to relieve either physical or non-physical pain (Fig 3). Physical pain simply refers to bodily pain often arising from the work one does. Research participants used tramadol to ease pain from their strenuous work. One of the interviewees, a painter who also often worked as a labourer and had used tramadol for a long time, noted:

Sometimes there are some works that I do which are very hard and when I take in that [tramadol] I don’t feel that pain. I can do the work over and over and I am not going to mess it up (W1).

Another interviewee, a welder and sprayer, noted that “with the drug when you are working you feel no pain and you do not feel weak, you do not feel anything, you just feel ok”. (T3) These descriptions, contrary to some of the media discourses, cohere with other literature on drug use in Africa that suggest that many young people use drugs such as cannabis for “deadening the pain of difficult work or alleviating the boredom of repetitive activities” [57]. In this sense, one of the main reasons why young Ghanaians use tramadol is to reduce physical pain connected to work.

Many young people also explained that they used tramadol to ease non-physical pain. In these cases, the drug is used as a way of navigating the condition of youth itself or to help deal with life struggles such as the death of a parent, while others use it to feel a sense of hope or to deal with boredom. This kind of pain is similar to that described by Weiss [58] in *The Barber in Pain*. It is pain that is not necessarily bodily but emerges from social structures. The youth described by Weiss in the barber shops of Arusha, Tanzania did not necessarily turn to drugs to deal with the pain of feeling marginalised but turned to music and work, and for them pain “presents an affirmation of one’s ongoing confrontation with obstacles, a process of grappling with life’s troubles that define existence itself” [58]. Additionally, while pain indexes social suffering it can also foster solidarities, where people “share and distribute pain” as Fullwiley [59] found for sickle cell sufferers in Senegal.

Many of the participants I interviewed described non-physical pain related to dropping out of school, losing a parent, not having a stable job or simply not feeling that the transition to adulthood was working out as they would have hoped. They often explained that apart from pain in the body there was “another pain” in their life for which tramadol was a panacea. Consider the following statement from a 27-year-old participant who had dropped out of school after completing his junior high school education because his parents could not afford to pay his school fees. He argued that tramadol gave him a certain ‘feeling’ he wanted to hold on to:

It gives you a certain feeling. It gives you vim. It makes you feel like you can still make it. You may see your colleague riding a motorcycle past and if you look at them you can have the feeling that perhaps even today you could make it. Personally, for me what used to happen was that whenever I would see a colleague like that it always gave me a certain vim to work more while having the same mood (T3)

Similarly, another participant described how his parents’ divorce coupled with poverty and feelings of dislocation led him to drug use:

The reason why I’m taking [tramadol], I’m looking back, it was because of poverty that the disagreement came between my mother and father, but if I become rich, I can solve that disagreement. If I get money… the money I have, if it increases, then the pain can diminish in my heart, because both of them are aged now. (T4)

According to Honwana [43], many young people in Africa today are confronted with waithood, “a prolonged and uncertain stage between childhood and adulthood that is characterised by inability to enter the labour market and attain the social markers of adulthood.” This reality is a by-product of global neoliberal processes that have made work elusive in many parts of Africa [60] causing alienation among many young people who can only eke out an existence. The evidence from the interviews suggests that many young people experience waithood—or even ‘boredom’ as described by Masquelier [61] for young people in Niger—in a manner that predisposes them to the use of tramadol as a coping mechanism. Like the youth in Dar es Salaam [62] who turn to heroin to deal with the difficult and prolonged transition to adulthood, the Ghanaian youth I interviewed have turned to tramadol to deal with some of the challenges of being young in Ghana today. In this sense their drug use partly represents a response to pain, waithood and the condition of youth.

### 4.4 Beyond pain

While most of this article has focused on pain and moral discourses, there are important variations in the contexts of tramadol use and interviewees also highlighted issues beyond pain. Some of these issues have been examined in extant literature on tramadol use in Ghana [14,16,17]. Briefly, reasons discussed by interviewees for tramadol use beyond pain were desires for pleasure or to alter one’s mood, the use of tramadol as an antidote to boredom, a desire not to appear lazy in the workplace and the use of tramadol as a form of sociality and to gain a sense of belonging.

Almost every participant indicated that they were aware of the unfavourable societal views of tramadol users and expressed a desire to be understood and seen as ‘normal’ people. Indeed, contrary to media representations of those who use tramadol as simply feckless and irresponsible, many youths expressed fears about the possibility of becoming addicted to tramadol. They acknowledged the dangers of addiction and balanced these with the pleasure tramadol gives and or the utility of the drug in improving their ability to function in the workplace. When asked why he used tramadol, one of the respondents noted that it played the dual role of making him happy and not feel lazy:

If I take that one [tramadol], I just have happiness, if am working, am just fine, I will not be having laziness in me or be sleepy. (T1)

The only female respondent similarly described how tramadol made her feel a sense of ‘calmness’. She noted that while this calmness was desired, she observed she needed to increase her dosage to experience this emotion and hinted at fears of addiction. She noted in her interview:

It makes me feel really calm. I sleep, by the time I wake everything is ok. If you get to this stage like my body, get[s] use[d] to the 100mg and we’ve had to increase it though it is not good, I think! For my system, for my own good. (A1)

Many youths highlighted fears of negative societal perceptions and of addiction. In some cases, fear of addiction was seen as a possible reason to quit the use of tramadol. When one participant was asked if he would quit using tramadol he responded:

Why I will stop using tramadol is that you can get addicted and when you don’t get it to take you may fall ill. The drug may also drain your body fluid (T4)

These fears of addiction coupled with worries about negative societal views of those who use tramadol were highlighted by interview respondents. One responded particularly noted how her mother and people in drug stores were taken aback when they realised she used tramadol:

They think everyone abuse it, like they just look at you in some certain way like, ‘she looks okay, why is she requesting for tramadol?’ There’s been an impression on the use of tramadol, that people that use it abuse it. And then they take it in larger doses. I do but I don’t take it that much, but people can take 200gm of it just for, I don’t know why they do that, so it causes that perception. In fact, even home, if am taking the medicine and say oh mommy this is tramadol. I am taking tramadol; she will say like eiii [exclamation] you’re taking tramadol for what? (A1)

The worries about the possibility of addiction as well as anxieties regarding negative societal perceptions of those who use tramadol were seen by some as possible reasons to quit using the drug. Beyond the moralising discourses described above such fears are partly justified because extant research shows that the public often has unfavourable views of those who use drugs when compared for example to people with (other) mental illnesses [63]. The factors described (pleasure, boredom avoidance, a desire not to appear lazy etc.) above while not directly related to pain also influenced tramadol use and highlight the complex nature of use among youth. Fears regarding addiction and awareness of negative societal views of tramadol users all highlight the humanity of tramadol users and reveals them to be young people seeking to find their place in the world rather than criminals or morally bankrupt people as represented by some in the media.

## 5. Conclusion

There is little doubt that the abuse of any drug can be dangerous and associated with addiction. Nonetheless dealing with the challenges of drug abuse among youth in marginalised contexts should be guided by a “harm reduction approach” [64] rather than the “tough on crime and anti-drug” [26] approach that has been adopted in Ghana thus far. In March 2020 Ghana signed the Narcotics Control Commission Bill [65] into law, signifying a major step towards viewing drug use and addiction as public health concerns rather than criminal justice problems. This shift is likely a move towards harm reduction and has the potential to play an important role in responding to drug use among Ghanaian youth.

Interpreted more broadly, tramadol is one of many ways Ghanaian youth are responding to social marginalisation. Like their predecessors who alarmed colonial authorities by “rebelling” [66], using tramadol is a way of navigating difficult social and economic conditions as well as coping with the prolonged transition to adulthood. In early May 2021, the Twitter hashtag #fixthecountrynow was used by several young Ghanaians to register their displeasure at the ongoing economic and social difficulties facing the country. Within a couple of weeks many young people were tweeting about bad roads, *dumsor* (power outages), water crises and other socioeconomic problems. The rise in tramadol use should be understood at least in part, as a reaction to economic realities created by decades of structural adjustment and neoliberal economic policies [9] that limit opportunities for decent work thus forcing young people to use drugs. These reactions merit structural intervention to improve the social determinants of health and to curb the potential dangers of addiction by youth. An important reason for opioid use among Ghanaian and West African youth is pain rather than immorality as portrayed by authorities and this calls for a rethinking of the sorts of solutions prescribed for dealing with the challenge of opioid use. Additionally, young people sometimes use tramadol in a non-problematic fashion thus necessitating proper engagement with youth as the country works on responding to the use of tramadol and other drugs.

## Supporting information

S1 Checklist COREQ checklist. (DOCX)

S1 Table Combined analysis and codes. (DOCX)

S1 Text Interview questions. (DOCX)

S2 Text Inclusivity in global research. (DOCX)
