# Supplementary material for: Where is the pain? A qualitative analysis of Ghana’s opioid (tramadol) ‘crisis’ and youth perspectives
Source: PLOS Glob Public Health. 2022 Dec 21;2(12):e0001045. doi: 10.1371/journal.pgph.0001045 (PMC10021380; doi:10.1371/journal.pgph.0001045)
Supplement: S1 Checklist — (DOCX) [file pgph.0001045.s001.docx]

COREQ Guidelines

|  |  | Reference in manuscript |
| --- | --- | --- |
| **Domain 1: Research team and reflexivity**  Personal Characteristics | 1. Interviewer/facilitator | Interviews were conducted by me and my positionality and personal characteristics are described under the section on reflexivity. Please see lines 251-258 |
|  | 1. Credentials |  |
|  | 1. Occupation |  |
|  | 1. Gender |  |
|  | 1. Experience and training |  |
| Relationship with participants | 1. Relationship established | Interviewees were recruited through a research poster and personal contacts between March and May 2021 and relationships were built through establishing rapport since I am myself a Ghanaian youth. Please see lines 251-258 |
|  | 1. Participant knowledge of the interviewer |  |
|  | 1. Interviewer characteristics |  |
| **Domain 2: study design**  Theoretical framework | 9. Methodological orientation and Theory | Moral panic and structural violence theories (lines 162-187) guided analysis and the qualitative methodology used was case study with thematic and discourse analysis (lines 192-195) |
| Participant selection | 10. Sampling | 15 participants were purposively sampled and non dropped out throughout the study. Interviews were conducted via telephone between March and May 2021 (please see lines 203-220) |
|  | 11. Method of approach |  |
|  | 12. Sample size |  |
|  | 13. Non-participation |  |
| Setting | 14. Setting of data collection | Participants joined the phone interviews from home and were alone during the interviews (lines 204-205). Participants were mostly male except for one female among the 10 youth who used tramadol (lines 211-214). Stakeholders comprised of 2 females and 2 males (lines 221-228). Youth were aged 19-35 (line 214) |
|  | 15. Presence of non-participants |  |
|  | 16. Description of sample |  |
| Data collection | 17. Interview guide | The interviews were conducted using a semi-structured interview guide (lines 216-220) and audio recorded, transcribed and imported into NVivo (line 205). 15 interviews were considered appropriate for saturation given emerging similarity of responses (lines 206-210). |
|  | 18. Repeat interviews |  |
|  | 19. Audio/visual recording |  |
|  | 20. Field notes |  |
|  | 21. Duration |  |
|  | 22. Data saturation |  |
|  | 23. Transcripts returned |  |
| **Domain 3: analysis and findings**  Data analysis | 24. Number of data coders | All data were coded by me using the six-stage approach suggested by Braun and Clark lines 242-247. Themes emerged from combining categories and based on coded interview extracts. The theming process is also discussed in lines 242-247. All analysis was done using NVivo 12 and a synthesised member check was done with four participants (lines 248-250) |
|  | 25. Description of the coding tree |  |
|  | 26. Derivation of themes |  |
|  | 27. Software |  |
|  | 28. Participant checking |  |
| Reporting | 29. Quotations presented | Quotations are presented throughout the manuscript and participants are numbered based on sequence of interview and location (line 230-232). Illustrative quotes reveal the main finding that pain is important to understand tramadol although moral discourses have been more prominent in media reports. Three main themes are discussed throughout the manuscript including Youth (lines 277-315), Moral failure (316-385), Pain (lines 386-486) and the minor theme of issues beyond pain (lines 488-539). |
|  | 30. Data and findings consistent |  |
|  | 31. Clarity of major themes |  |
|  | 32. Clarity of minor themes |  |
