# Supplementary material for: Where is the pain? A qualitative analysis of Ghana’s opioid (tramadol) ‘crisis’ and youth perspectives
Source: PLOS Glob Public Health. 2022 Dec 21;2(12):e0001045. doi: 10.1371/journal.pgph.0001045 (PMC10021380; doi:10.1371/journal.pgph.0001045)
Supplement: S1 Table — (DOCX) [file pgph.0001045.s002.docx]

Media Discourse Codes

| Name | Files | References |
| --- | --- | --- |
| Causes | 0 | 0 |
| Economic causes | 0 | 0 |
| Economy | 1 | 2 |
| Poverty | 1 | 1 |
| Profit | 1 | 1 |
| Structural conditions | 1 | 1 |
| Individual causes | 0 | 0 |
| Athletes | 1 | 1 |
| For Fun | 1 | 7 |
| Misperception | 1 | 1 |
| Peer pressure | 1 | 6 |
| To help study | 1 | 2 |
| Political causes | 0 | 0 |
| Governance | 1 | 2 |
| Social and Cultural Causes | 0 | 0 |
| Lack of Pharmacists | 1 | 1 |
| Natural pain | 1 | 2 |
| Over-prescription | 1 | 1 |
| Social media | 1 | 2 |
| Socioeconomic context | 1 | 1 |
| Working harder | 1 | 5 |
| Contract awarded | 1 | 2 |
| Drug routes | 1 | 2 |
| Cartels | 1 | 3 |
| ECOWAS | 1 | 2 |
| Unapproved routes | 1 | 6 |
| Effects on individuals | 0 | 0 |
| addiction | 1 | 5 |
| Death | 1 | 3 |
| Get high | 1 | 1 |
| Headache | 1 | 1 |
| physical effects | 1 | 12 |
| Poverty traps | 1 | 1 |
| Effects on society | 0 | 0 |
| Accidents | 1 | 1 |
| Armed robbery | 1 | 1 |
| Crime | 1 | 3 |
| Family income | 1 | 1 |
| Health system | 1 | 4 |
| Lost family members | 1 | 1 |
| Low productivity | 1 | 5 |
| Macro level | 1 | 1 |
| National image | 1 | 1 |
| Prostitution | 1 | 2 |
| Security | 1 | 2 |
| Social liability | 1 | 1 |
| Terrorism | 1 | 1 |
| Moral Panic | 0 | 0 |
| Act now | 1 | 4 |
| Crisis | 1 | 8 |
| Dilemma | 1 | 1 |
| End of the drama | 1 | 2 |
| Fentanyl | 1 | 1 |
| Few people use drug | 1 | 1 |
| From Marijuana to Tramadol | 1 | 6 |
| Future of the country | 1 | 8 |
| International Panic | 1 | 1 |
| Many students and Youth use drugs | 1 | 34 |
| Move to cough syrup | 1 | 1 |
| National enemies | 1 | 1 |
| Public health crisis | 1 | 1 |
| Rise in metamphetamine production | 1 | 1 |
| Rising usage | 1 | 7 |
| Slapped Senior housemaster | 1 | 1 |
| Social canker | 1 | 6 |
| The medicine is still on sale | 1 | 1 |
| Tramadol trend | 1 | 2 |
| Regulatory responses | 0 | 0 |
| Ministry reviewing laws | 1 | 1 |
| Tramadol reclassified | 1 | 2 |
| Representation of Tramadol | 0 | 0 |
| Innocuous but dangerous | 1 | 1 |
| Seizures | 0 | 0 |
| Truck from Ghana drugs from China | 1 | 1 |
| West Africa | 1 | 1 |
| Solutions | 0 | 0 |
| Advise | 1 | 7 |
| Context specific | 1 | 1 |
| Counselling | 1 | 1 |
| Education | 1 | 10 |
| Individual responsibility | 1 | 1 |
| Inspire | 1 | 2 |
| Law Abiding citizens | 0 | 0 |
| Laws | 1 | 2 |
| Morals | 1 | 12 |
| Moral code | 1 | 1 |
| Multi Sectoral Collaboration | 1 | 4 |
| Planting for jobs | 1 | 1 |
| Prescription use only | 1 | 1 |
| Prosecution | 0 | 14 |
| Regional Drug control | 1 | 1 |
| Regulate Media | 1 | 1 |
| Regulation | 1 | 7 |
| Rehab and training | 1 | 5 |
| Seizure | 1 | 15 |
| Police Swoops | 1 | 18 |
| Traditional and religious authorities | 1 | 12 |
| Stakeholders | 0 | 0 |
| Pharmacy silence | 1 | 1 |
| Insecurity | 0 | 0 |
| Pain | 1 | 1 |
| Youth | 1 | 13 |
| Burden | 1 | 1 |
| Contribute quota | 1 | 1 |
| Dreams | 1 | 1 |
| Guide the youth | 1 | 2 |
| Save them | 1 | 4 |
| Tertiary students | 1 | 1 |
| Youth development | 1 | 5 |
| Users | 0 | 0 |
| Kaya Yei | 1 | 1 |
| laborers | 1 | 1 |
| Why problem persists | 0 | 0 |
| Political interference | 1 | 1 |

**Patterns of reportage and framing**

1. Individual and Non individual causes
2. Effects on Individual
3. Effects on society
4. Moral panic and youth immorality
5. Solutions to the problem

| Individual and Non individual causes | Effects on Individual | Effects on society |
| --- | --- | --- |
| - Economic causes/Economy - Poverty - Profit - Structural conditions - Lack of Pharmacists - Over-prescription - Social media - Working harder - Poverty traps - Pharmacy silence - Athletes - For Fun - Misperception - Peer pressure - To help study | - Addiction - Death - Get high - Headache - physical effects - Dreams - Youth development | - Accidents - Armed robbery - Crime - Family income - Health system - Lost family members - Low productivity - Macro level - Terrorism - Social canker - Contribute quota |
| Moral panic and youth immorality | Solutions to the problem |  |
| - Accidents - Armed robbery - Crime - Lost family members - National image - Prostitution - Security - Social liability - Act now - Crisis - Dilemma - End of the drama - Fentanyl - From Marijuana to Tramadol - Future of the country - Many students and Youth use drugs - Move to cough syrup - National enemies - Public health crisis - Rise in methamphetamine production - Rising usage - Slapped Senior housemaster - Innocuous but dangerous - Youth - Burden | - Truck from Ghana drugs from China - Ministry reviewing laws - Tramadol reclassified - Advise - Context specific - Counselling - Education - Individual responsibility - Inspire - Law Abiding citizens - Laws - Morals - Moral code - Multi Sectoral Collaboration - Planting for jobs - Prescription use only - Prosecution - Regional Drug control - Regulate Media - Regulation - Rehab and training - Seizure - Police Swoops - Traditional and religious authorities - Guide the youth - Save them |  |

**Key discourse from media analysis**

Main: Tramadol use as problem of individual moral failure

Minor: solutions lie in advice and some regulation

**Individual interview thematic analysis codes**

| Name | Files | References |
| --- | --- | --- |
| Accessing without prescription | 1 | 1 |
| Addiction and usage | 3 | 6 |
| Addicted | 2 | 5 |
| Fear of addiction | 3 | 4 |
| Fears at initial usage | 1 | 2 |
| Cessation of consumption conditions | 0 | 0 |
| New and better job | 2 | 5 |
| Office job | 4 | 7 |
| Discernment and discrimination | 0 | 0 |
| Bad versus good tramadol | 1 | 2 |
| Better than them | 3 | 6 |
| Calming weed | 0 | 1 |
| Non work tramadol users | 1 | 1 |
| Weed enlightens | 2 | 3 |
| Dosage | 3 | 10 |
| Dropping out of school | 2 | 7 |
| Low earnings from work | 2 | 5 |
| Friend gave drug | 1 | 3 |
| Friendship ends after quitting | 1 | 1 |
| Future plans | 0 | 0 |
| Learn truck driving | 1 | 1 |
| Become soldier | 2 | 5 |
| Help for addiction | 0 | 0 |
| Low income | 3 | 7 |
| Learning to use drugs | 0 | 0 |
| Brothers influenced to use | 1 | 1 |
| Worries and painful thoughts | 4 | 12 |
| Length of usage | 2 | 3 |
| Mode of ingestion | 0 | 0 |
| Injection | 1 | 1 |
| Other drugs | 0 | 0 |
| Cocaine | 1 | 1 |
| Marijuana | 3 | 6 |
| Marijuana over tramadol | 1 | 1 |
| Marijuana Tea | 1 | 1 |
| Plan to quit marijuana | 1 | 1 |
| Use drug with others | 2 | 3 |
| Problematic use | 0 | 0 |
| Bad mixing | 2 | 3 |
| combining with other drugs | 2 | 1 |
| Make it prescription only | 1 | 1 |
| Rehabilitation | 0 | 0 |
| no knowledge of rehab opportunity | 2 | 6 |
| Sex | 0 | 0 |
| Not for sex | 3 | 5 |
| Social perception of users | 0 | 0 |
| fear of disclosure | 1 | 6 |
| Futureless | 3 | 7 |
| Lazy | 4 | 4 |
| Looked down on | 2 | 6 |
| Misunderstood users | 4 | 5 |
| Useless | 2 | 6 |
| Will go mad | 2 | 3 |
| Sources | 0 | 0 |
| Drug dealers | 2 | 2 |
| Drug store | 3 | 6 |
| Used not always | 3 | 6 |
| Why use drug | 0 | 0 |
| Broken family | 1 | 7 |
| Busy lifestyle | 3 | 8 |
| Calmness | 3 | 8 |
| Hustler lifestyle | 4 | 7 |
| Juggling multiple responsibilities | 2 | 6 |
| No boredom | 1 | 1 |
| Non-physical pain | 0 | 0 |
| emotional pain | 3 | 12 |
| Anxiety | 4 | 6 |
| Loneliness | 5 | 8 |
| precariousness | 6 | 17 |
| Worries and thoughts | 4 | 8 |
| Not to be seen as lazy | 1 | 5 |
| Pain Killer | 5 | 1 |
| Physical pain | 3 | 13 |
| Pleasure | 0 | 0 |
| Happy mood | 4 | 4 |
| Poverty | 3 | 7 |
| Sickness | 3 | 8 |
| stress | 5 | 9 |
| Work | 6 | 2 |
| Long hours | 2 | 9 |
| Physically demanding | 4 | 8 |
| Strength to work | 2 | 8 |
| ‘Tiredness cure’ | 2 | 12 |
| Work faster | 5 | 8 |
| Work more than others | 2 | 6 |
| Work stress | 2 | 4 |

Combined codes to form categories

| Access and addiction to tramadol | Societal views of tramadol users | Individual experiences and family level causes of tramadol use |
| --- | --- | --- |
| - Accessing without prescription - Addiction and usage - Addicted - Fear of addiction - Fears at initial usage - Dosage - Other Mode of ingestion - Injection - Other drugs - Cocaine - Marijuana - Marijuana over tramadol - Marijuana Tea - combining with other drugs - Drug dealers - Drug store | - Discernment and discrimination - Bad versus good tramadol - Better than them - Social perception of users - fear of disclosure - Lazy - Looked down on - Will go mad | - Cessation of consumption conditions - New and better job - Office job - Dropping out of school - Friend gave drug - Plan to quit marijuana - Problematic use - Bad mixing - Broken family |
| Physical pain and tramadol use | Emotional pain and tramadol use | Pleasure, Friendship and tramadol use |
| - Low earnings from work - Low income - Busy lifestyle - Hustler lifestyle - Juggling multiple responsibilities - No boredom - Pain Killer - Physical pain - Poverty - Sickness - Stress - Long hours - Physically demanding - Strength to work - ‘Tiredness cure’ - Work faster - Work more than others | - Worries and painful thoughts - Futureless - Looked down on - Misunderstood users - Useless - Broken family - Loneliness - Worries and thoughts - Stress - Work stress - Anxiety - Precariousness | - Calming weed - Non work tramadol users - Weed enlightens - Friendship ends after quitting - Use drug with others - Sex - Not for sex - Calmness - Pleasure - Happy mood |
| Youth and futures | Solutions to the problem |  |
| - Future plans - Learn truck driving - Become soldier - Futureless | - Help for addiction - Make it prescription only - Rehabilitation - no knowledge of rehab opportunity |  |

**Categories**

1. Access and addiction to tramadol
2. Societal views of tramadol users
3. Individual experiences and family level causes of tramadol use
4. Physical pain and tramadol use
5. Emotional pain and tramadol use
6. Pleasure, friendship and tramadol use
7. Youth and Future
8. Solutions to the problem

**Themes**

1. **Youth**
2. **Pain**
3. **Negative societal views**
4. **Beyond pain**

| 1. **Youth** | Youth plans for the future and feeling dislocated |
| --- | --- |
| 1. **Pain** | Main theme focused on pain and its materiality. Subdivided into physical and non-physical and developed into framework |
| 1. **Moral judgement** | Negative societal views on those who use tramadol (combined with media analysis on morality) |
| 1. **Beyond pain** | Other important perspectives beyond pain - pleasure, calmness and sociality connected to processes and practices of use |
