# Supplementary material for: Where is the pain? A qualitative analysis of Ghana’s opioid (tramadol) ‘crisis’ and youth perspectives
Source: PLOS Glob Public Health. 2022 Dec 21;2(12):e0001045. doi: 10.1371/journal.pgph.0001045 (PMC10021380; doi:10.1371/journal.pgph.0001045)
Supplement: S1 Text — (DOCX) [file pgph.0001045.s003.docx]

**Interview questions**

1. Tell me about yourself (age, gender, occupation, other demographic indicators and brief life history)
2. Tell me about when you started using tramadol

- Please clarify if tramadol was prescribed by a health worker or not

1. How often do you take tramadol and why?

- Tell me about how you started using tramadol
- Tell me more about why you use the drug?

1. What would it take for you to stop using tramadol?
2. How do people view/perceive tramadol consumption and those who use the drug?
3. Tell me more about how you access tramadol?

- Is it from a drug store?
- Is it from the black market?

1. Beyond your personal choice, can you think of ‘social factors’ that make you use tramadol or the other drugs you mentioned in this interview?
2. Tell me more about the people with whom you use the drug?
3. Tell me more about any supports available to you if you wanted to stop using tramadol?

- Would you like rehabilitation? Do you know how to find help?

1. Do you have hope for the future as Ghanaian youth?

- Ten Years from now what do you think you will be doing?

**Stakeholder Questions**

1. Tell me a little about yourself and the context of your work
2. How did Tramadol become so topical in Ghana

- What is the story of the rise in consumption?

1. What social factors are driving (or drove) tramadol consumption?
2. What political factors are driving (or drove) tramadol consumption?
3. What economic factors are driving (or drove) consumption?
4. What have been some of the policy responses to tramadol?
5. How successful and appropriate have policy responses been?
6. Apart from police arrests/education/media campaign is there another way to deal with the rise in consumption?
7. What have been the effects of current policy responses?
8. What do you think is the future of the tramadol ‘crisis’?
9. How equipped are the health and social service systems to deal with the crisis?
